# Dry-Adhesive Microstructures for Material Handling of Additively Manufactured and Deep-Rolled Metal Surfaces with Reference to Mars

**DOI:** 10.3390/ma16114170

**Published:** 2023-06-03

**Authors:** Nicole Mensching, Mirja Louisa Krüger, Askar Kvaratskheliya, Daniel Meyer, Kirsten Tracht, Ilya Okulov, Lutz Mädler

**Affiliations:** 1 Center for Materials and Processes (MAPEX), University of Bremen, Bibliothekstr. 1, 28359 Bremen, Germany; mensching@iwt-bremen.de (N.M.); krueger@bime.de (M.L.K.); askar@iwt.uni-bremen.de (A.K.); tracht@bime.de (K.T.); i.okulov@iwt.uni-bremen.de (I.O.); lmaedler@iwt.uni-bremen.de (L.M.); 2 Leibniz Institute for Materials Engineering IWT, Badgasteiner Straße 3, 28359 Bremen, Germany; 3 Bremen Institute for Mechanical Engineering (bime), University of Bremen, Badgasteiner Str. 1, 28359 Bremen, Germany; 4 Faculty of Production Engineering, University of Bremen, Badgasteiner Straße 2, 28359 Bremen, Germany

**Keywords:** surface properties, dry-adhesive microstructures, deep-rolling, additive manufacturing

## Abstract

Once on Mars, maintenance and repair will be crucial for humans as supply chains including Earth and Mars will be very complex. Consequently, the raw material available on Mars must be processed and used. Factors such as the energy available for material production play just as important a role as the quality of the material that can be produced and the quality of its surface. To develop and technically implement a process chain that meets the challenge of producing spare parts from oxygen-reduced Mars regolith, this paper addresses the issue of low-energy handling. Expected statistically distributed high roughnesses of sintered regolith analogs are approximated in this work by parameter variation in the PBF-LB/M process. For low-energy handling, a dry-adhesive microstructure is used. Investigations are carried out to determine the extent to which the rough surface resulting from the manufacturing process can be smoothed by deep-rolling in such a way that the microstructure adheres and enables samples to be transported. For the investigated AlSi10Mg samples (12 mm × 12 mm × 10 mm), the surface roughness varies in a wide range from Sa 7.7 µm to Sa 64 µm after the additive manufacturing process, and pull-off stresses of up to 6.99 N/cm^2^ could be realized after deep-rolling. This represents an increase in pull-off stresses by a factor of 392.94 compared to the pull-off stresses before deep-rolling, enabling the handling of even larger specimens. It is noteworthy that specimens with roughness values that were previously difficult to handle can be treated post-deep-rolling, indicating a potential influence of additional variables that describe roughness or ripples and are associated with the adhesion effect of the microstructure of the dry adhesive.

## 1. Introduction

Since new products have to meet high requirements regarding performance and lifetime, innovations are always based on the use of materials as pure as possible. Therefore, making things “as good as possible” instead of “as good as necessary” is the driver of current developments. However, the resources available on Earth are limited, and with them the possibility to continue striving for the optimum in material purity in the future. Thus, recycling is becoming increasingly important, although this may involve restrictions with regard to the material’s performance. The degree of required material purity may vary depending on the specific application [1,2].

The limited availability of pure resources and the need to produce usable, application-oriented components with the available resources link Earth and Mars with each other. Assuming future settlement on Mars, maintenance and repair will be crucial for humans as supply chains involving Earth and Mars will be very complex. The available resources in terms of raw materials and energy must be sufficient to allow things to function. The question, therefore, arises to what extent the regolith available on Mars (among other contents consisting of various oxide compounds, such as SiO_2_, MgO, Al_2_O_3_, FeO, and CaO) [3] can be used for the generation of components? The optimal proportion of pure materials required for synthesis and the maximum acceptable level of impurities, such as regolith, in a material mixture to ensure sufficient quality for producing construction or spare parts remains unclear.

The limited resources on Mars require extremely efficient use of energy, raw material, equipment, and also production space. Since all machines and manufacturing equipment have to be initially transported from Earth, every component has to be optimized. This also includes the handling of workpieces and parts comprised of raw materials available on Mars. The handling has to be machine-based using robotic handling systems in order to realize autonomous production. Efficient part handling is directly connected to the required area on the part’s surface when it comes to the use of dry-adhesive microstructures. For manufacturing chains, also the overarching process efficiency and possible synergies between different processes have to be considered when planning to manufacture parts on Mars. Due to the low energy available on Mars, energy-efficient handling of workpieces and parts is necessary. Dry-adhesive microstructures are particularly well suited for low-energy handling because no energy is needed to grip and hold the workpieces and low energy is needed to release them. However, the dry-adhesive microstructures require a distinct surface area to adhere, and the surface properties in this area affect the formation of the adhesive force. Since the workpieces and parts created from impure materials on Mars will not initially have suitable surface properties, processes must be provided to positively modify the surfaces.

**Figure 1:**
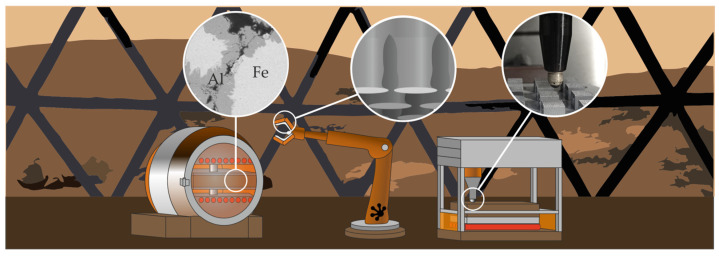
Scheme of the future planned process chain, including sintering (**left**), handling (**middle**), and strain hardening by deep-rolling (**right**).

These aspects are approached with means of a process chain currently under development (see Figure 1), whose core is formed by a sintering and deep-rolling as a surface treatment process. Following the rolling process, the question of handling possible (semi-finished) products arises. Taking into account the low energy available on Mars [4] over the entire process chain, the heat of the sintering process should, in perspective, benefit the rolling process (lower forces required, and higher depth effect with warmer samples [5]). The handing should be conducted using dry-adhesive microstructures, which can adhere to surfaces without additional forces [6]. Alternative gripping technologies, such as vacuum grippers, magnetic grippers, and centric grippers, require energy for gripping and also during the entire handling process rather than grippers with dry-adhesive microstructures. Jiang et al. have already used dry-adhesive microstructures in their experiments as gripper technology to handle objects [7]. In addition, dry-adhesive microstructures have been used to collect space debris in space, which suggests that it is possible to use the microstructures on Mars [8]. However, the reduction of the adhesive force by contaminants, such as dust or wear residues, is problematic, which would render the exploration of different cleaning options interesting [9].

Since it is already known that the adhesion of dry-adhesive microstructures depends on the surface properties of the object to be handled [10], the following questions concerning the handling are addressed in this work:

- Is the handling of samples of high surface roughness, such as those resulting from sintering or additive manufacturing processes, possible without further machining by a rolling process, and, if so, what is the maximum pull-off stress?
- How large is the improvement in adhesion of the dry-adhesive microstructures due to a rolling process?
- When increasing the specimen size, what proportions of the surfaces should be rolled to ensure adhesion for the time required for handling?

In the following sections, the individual steps of the process chain are regarded in detail considering the conception of an autonomous production on Mars.

Previous research demonstrated the feasibility of metal production using in situ resource utilisation (ISRU), most commonly in the form of a metal powder produced by an electrochemical reaction [11,12,13]. Several structural metals that could be derived from Martian soil are under consideration, such as Fe, Al, Si, and Mg, which potentially allow the production of the metal investigated in this work [12,14,15]. In this study, the PBF-LB/M method is employed to replicate distinct surface roughness characteristics. The rationale for using this method was based on the similarity between the surface roughness parameters of additive manufacturing and those of conventional sintering methods [16,17]. Furthermore, additive manufacturing itself is a potential method for the in situ utilization of regolith for the production of parts on the moon or on Mars [18,19].

Since regolith sintering is still a challenge because of the high required temperature for the process, the investigations in this work address the questions posed by using AlSi10Mg samples of different roughness produced utilizing PBF-LB/M [20]. It is assumed that the surface properties are comparable to those of sintered specimens and that the knowledge gained can be transferred to subsequent investigations. The additive technologies are extremely promising and the use of this method will be necessary as the production of complex specific parts to replace breakage will be the only option. A complex production as on Earth will not be available on Mars and it will be impossible to transport the required replacement from Earth in a short time [21,22].

Hydrostatic deep-rolling is used as mechanical surface treatment. Investigations by Denkena et al. on cast AlSi10Mg show good formability of the surface using a spherical 6.35 mm tool (HG 6 by Ecoroll AG, Celle, Germany). With a deep-rolling pressure of up to 9 MPa, indentation depths of more than 8 µm and indentation widths of more than 800 µm are generated by the tool in single-track experiments [23]. It can be assumed that, comparable with the use of other materials, the depth effect of the process increases with increasing rolling pressure [24] or increased workpiece temperature [5], so that, in addition to influencing the surface, work hardening and residual compressive stresses result [25]. The fact that deep-rolling can be used to smooth the surface of PBF-LB/M has been demonstrated by previous work on 316L [26,27].

The mushroom-shaped microstructure of the dry-adhesive microstructures resembles the microstructure of the leaf beetle foot and not necessarily the microstructure of the gecko foot [28]. Due to the reversible adhesion properties through the Van der Waals forces, the dry-adhesive microstructures are used as grippers for various handling processes [6]. This quite new gripper technology works without electricity or compressed air and, therefore, can be used under a vacuum and when a low energy consumption is needed. When used as a gripper, surface roughness can be an obstacle to adhesion, as the intermolecular interactions occur over a short distance. Excessive roughness can, therefore, reduce the formation of the interactions [29] and the maximum holding force, depending on the object geometry and the material pairing. If the surface roughnesses becomes too large, the contact is broken and the maximum holding force is reduced [30].

Gorumlu et al. also found that the microstructures adhere better to smooth surfaces. In their experiments, the dry-adhesive microstructures stick with an adhesive force of 10 N/cm^2^ on a smooth surface and with an adhesive force of 1 N/cm^2^ on a rough surface with a quadratic mean Rq from 373 nm to 618 nm [10].

Bauer et al. also conducted investigations with the dry-adhesive microstructures. In the experiments, the dry-adhesive microstructures are pressed onto a flat, rough aluminium specimen. The adhesion in these experiments is 35% to 50% lower compared to experiments on a flat, smooth glass substrate [31].

In addition, the adhesion of the dry-adhesive microstructures is limited by the onset of buckling. Bauer et al. also found that the dry-adhesive microstructures adhere better and have higher pull-off stresses on wavy, rough surfaces than on flat-tip structures [31].

## 2. Materials and Methods

### 2.1. Additive Manufacturing of Samples Using Laser Powder Bed Fusion (PBF-LB/M)

Spherical gas atomized AlSi10Mg powder supplied by the company Tekna is used for PBF-LB/M in this study. The AlSi10Mg powder particles have an average diameter of 45 ± 10 μm. Their chemical composition can be found in Table 1.

**Table 1:** Initial chemical composition of the powder [32].

Al	Si	Mg	Fe	Ti	Mn	Zn	Others
85.00–87.00%	9.00–11.00%	0.20–0.45%	≤0.50%	≤0.15%	0.40%	≤0.10%	0.80–1.50%

**Figure 2:**
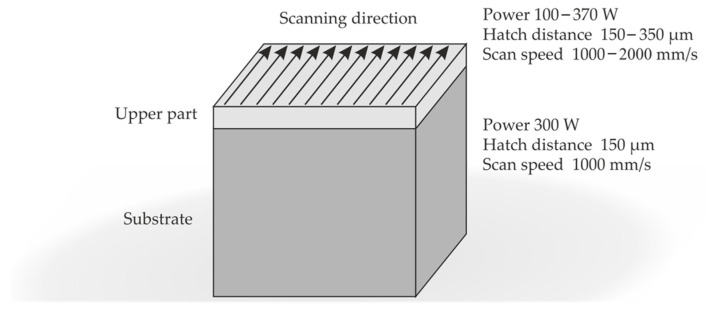
Visualisation of the strategy applied when printing and using parameters.

The samples in the form of parallelepipeds (12 × 12 × 10 mm), are additively manufactured by a PBF-LB/M process (SLM-125HL, by SLM Solutions, Lübeck, Germany). They consist of two parts, namely, substrate (lower part) and finishing (higher part). Both parts are fabricated using the laser-beam diameter of ∼70 μm and the powder layer thickness of 30 μm. The effective layer thickness resulting from printing is greater than the layer thickness to which the platform is lowered. This is why the layer density is lower than the average diameter of the powder particles [33]. The substrate part is manufactured using a laser power of 300 W, a hatch distance of 150 µm, and a scan speed of 1000 mm/s. In Figure 2, visualization of realized strategy could be found.

To obtain different mean arithmetic height Sa parameters, the finishing part (last three layers) is manufactured using different laser parameters. Specifically, the laser power is varied from 100 W to 370 W. The hatch distance is varied from 150 µm to 350 µm and the scan speed is varied from 1000 mm/s to 2000 mm/s. The additive manufacturing process is carried out under a high-purity argon atmosphere. The variation of the previously mentioned parameters is chosen following Maamoun, Xue et al., as they reached surface roughness between 5 µm and 14 µm [34].

### 2.2. Characterisation of the Surface

After the production of the samples using the PBF-LB/M process, the surfaces of the 24 samples, are examined with a 3D laser-scanning microscope using a 5× magnification (VK-X100K/X200K 3D laser-scanning microscope by Keyence Deutschland GmbH, Neu-Isenburg, Germany). The surface quality is determined according to DIN EN ISO 25178 with the analysis software “VK Analyse Modul” (version 3.8.0.0). The mean arithmetic height Sa is determined for all samples by evaluating the entire area of the microscope image using the mentioned ISO standard. Subsequently, 12 of the 24 samples are deep-rolled and then the mean arithmetic heights are determined again. To decide which samples are to be deep-rolled, the different surface parameters mean arithmetic height Sa, maximum height Sz, and the total height of the ripple Wz are compared between the specimen and matching pairs are formed, one sample of each pair was deep-rolled.

### 2.3. Deep-Rolling

**Figure 3:**
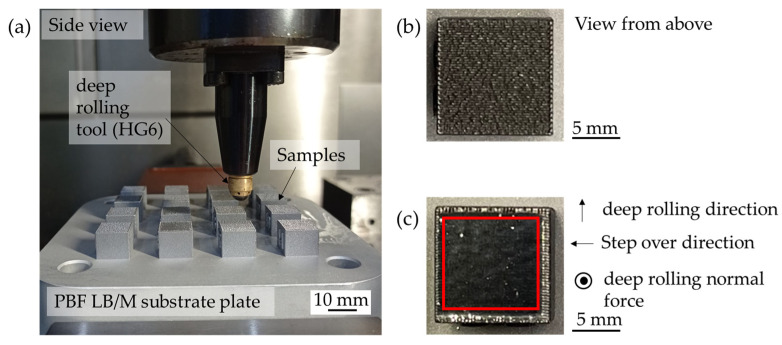
Experimental setup (**a**); sample before (**b**) and after deep-rolling an area of 10 mm × 10 mm (**c**).

Deep-rolling of the PBF-LB/M samples is performed on a three-axis CNC machining center (DMC 65V by Deckel Maho, Pfronten, Germany). The experimental setup can be seen in Figure 3a. A hydrostatic deep-rolling tool with a spherical ceramic tip HG 6 (diameter d_b_ = 6.3 mm) produced by Ecoroll (Ecoroll AG Werkzeugtechnik, Celle, Germany) is utilized to generate a deep-rolled area of 10 mm × 10 mm. During hydrostatical deep-rolling, the rolling tool is pressed on the surface with the deep-rolling normal force F_r_. The tool is then routed over the workpiece by the CNC machining centre. The deep-rolling direction is chosen perpendicular to the visible PBF-LB/M surface structure. Using a step over of 0.1 mm each area is generated by 100 single deep-rolling tracks. Figure 3b shows a sample before and Figure 3c a sample after deep-rolling. On the surface, the tool is flattening roughness spikes. Moreover, the chosen track stepover is much smaller than the contact width of the workpiece and the tool. Therefore, the resulting surface roughness after the deep-rolling process is significantly lower compared to the initial roughness. Due to the contact width of the deep-rolling tool and the process design focusing on the center of the tool the actual deep-rolled area of the specimens is slightly larger. Therefore, an area of 10 mm × 10 mm can be selected to test the dry-adhesive microstructure tapes without the effects of the non-deep-rolled outer area. In Figure 3c, the 10 mm × 10 mm area generated during the process can be seen marked red. In addition, it is visible that the actual deep-rolled area is larger by the indentation width of the deep-rolling tool.

The force measurement during the deep-rolling process is performed with a piezoelectric three-component dynamometer (Typ 9257 B, Kistler Instrumente GmbH, Sindelfingen, Germany) equipped with a Kistler charge amplifier Typ 5019 A. The deep-rolling pressure of 10 MPa, selected in accordance with Denkena et al. [23], resulted in a deep-rolling force F_r_ of 242.33 N ± 5.97 N. The process parameters and force measurement settings are summarized in Table 2. After deep-rolling, the specimens are electrolytically cut off the aluminum base plate for further investigations.

### 2.4. Method for Quantifying Adhesion

**Figure 4:**
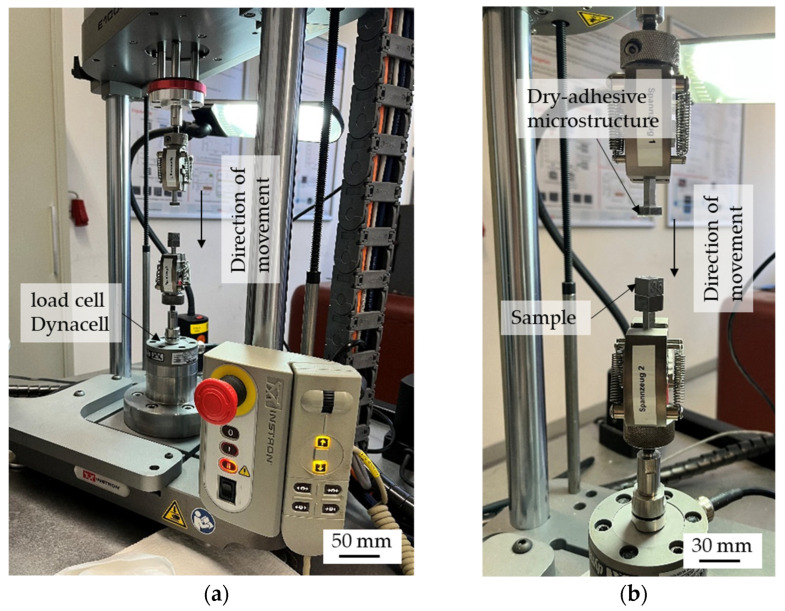
ElectroPuls^®^ E1000 testing machine (**a**) with the clamped adapters and the attached specimens or dry-adhesive microstructures (**b**).

The samples are cleaned in an ultrasonic bath (Bandelin Sonorex TK 52 by Bandelin electronic GmbH & Co. Kg, Berlin, Germany) with isopropanol for 15 min and then dried with compressed air so that they are free of dust and grease. An ElectroPuls^®^ E1000 testing machine (by Instron GmbH, Darmstadt, Germany) is used to determine the pull-off stress σ_pullmax_. The machine is equipped with the load cell Dynacell—Dynamic Load Cell ± 250 N (±56 lbf) (Instron GmbH, Darmstadt, Germany). The specimens and the dry-adhesive microstructures are attached to specially manufactured adapters and then clamped in the chucks of the testing machine. The testing machine and the clamped adapters with a sample and the dry-adhesive microstructure, respectively, can be seen in Figure 4a,b.

**Figure 5:**
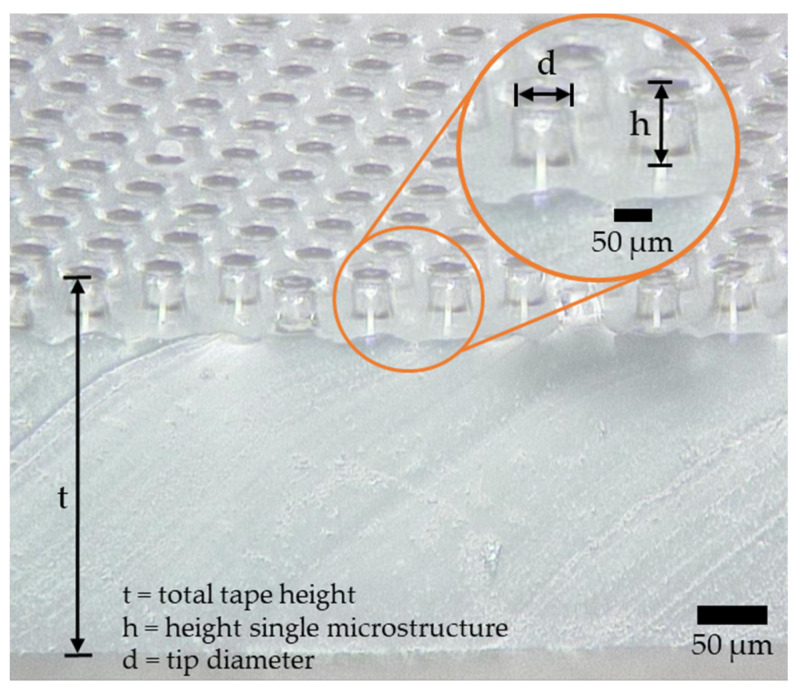
Laser-scanning microscope image of the dry-adhesive microstructure, taken by T. Brunkhorst, H. Dierks, H. Siesenis, and C. Tuitje.

As dry-adhesive microstructure tape, Gecko^®^ Nanoplast^®^ (Klettband Technik, Waldenbuch, Germany) is used for the experiments. This tape has a mushroom-shaped surface, whereby the adhesion between the samples and the dry-adhesive microstructures is realized by Van der Waals forces. In Figure 5, the dry-adhesive microstructures are shown in an image created with a laser-scanning microscope. A single mushroom head has an approximate tip diameter d of 38 µm and an approximate height h of 60 µm. The individual dry-adhesive microstructures are deposited on a silicone rubber film. The entire tape has a height t of 0.34 mm. The dry-adhesive microstructures can adhere to smooth and flat surfaces, even if they are wet, greasy, or soapy, without reducing the adhesive force. The Van der Waals forces that build up create electrostatic interactions that result in an intermolecular attraction force. The dry-adhesive microstructures can be used in a vacuum and can be removed without leaving any residue with low energy input. In addition, the dry-adhesive microstructures can be reused and cleaned with water [35]. The size of the dry-adhesive microstructure tape in each experiment is 10 mm × 10 mm. The sections of the tape are cut out with the help of a shaping punch.

A machining program is developed to determine the pull-off stress. The program moves the upper chuck down at a speed of 0.1 mm/s until the dry-adhesive microstructures touch the sample. Then the contact pressure is increased by 2 N/s until a contact pressure of 10 N is reached. This pressure is maintained for 10 s before the upper collet with the dry-adhesive microstructures is raised again at a speed of 0.1 mm/s and the pull-off stress can be measured.

For statistical validation, the pull-off stress is determined five times for each sample. A new dry-adhesive microstructure is used for each new adhesion and pull-off test in order to exclude defects due to wear or contamination. After each adhesion test, the PBF-LB/M samples are cleaned with ethanol to remove dirt or torn microstructures.

## 3. Results

### 3.1. Surface Roughness before and after Deep-Rolling

The surface of the PBF-LB/M samples is characterized using the 3D laser-scanning microscope (VK-X100K/X200K 3D laser-scanning microscope by Keyence, Deutschland GmbH, Neu-Isenburg, Germany) before and after deep-rolling. The images are evaluated according to DIN EN ISO 25178. The Sa values determined for the individual samples are shown in Table 3. It can be seen that the roughness of the samples is significantly reduced by the rolling process. Before deep-rolling, the maximum Sa value is 64 µm and the minimum Sa value is 7.7 µm. After deep-rolling, the maximum Sa value is 19.1 µm and the minimum Sa value is 0.3 µm. The mean arithmetic height Sa values after sample generation and deep-rolling can be compared with the results of Wielki and Meyer [26,27].

**Figure 6:**
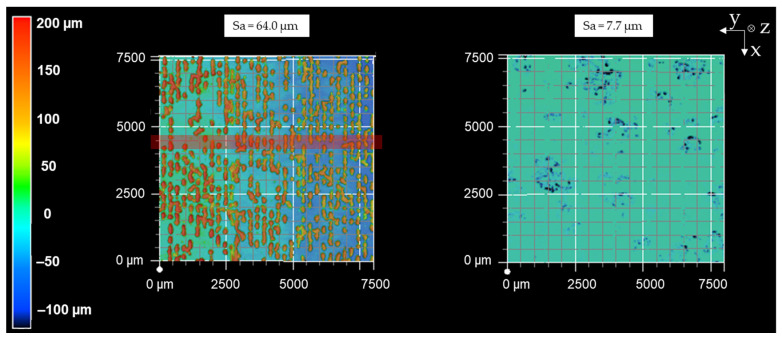
Surface topography before (**left**) and after (**right**) deep-rolling sample 19.

Depending on the surface roughness after the sample generation, the deep-rolling process was able to reduce the Sa values up to 98% (e.g., sample 5). Regarding the considered range of surface roughness, the potential for smoothing is higher with a higher initial roughness. Figure 6 exemplarily shows the surface topography of sample no. 19 before (Sa = 64.0 µm) and after deep-rolling (Sa = 7.7 µm). The results show that deep-rolling leads to a reduction in surface profile elevations, which is frequently observed after the specimen generation. Furthermore, a decrease in roughness over the entire surface area is observed following the rolling process. Reducing the roughness level allows more individual microstructures of the dry-adhesive tape to adhere to the surface and increases the maximum pull-off stress.

**Figure 7:**
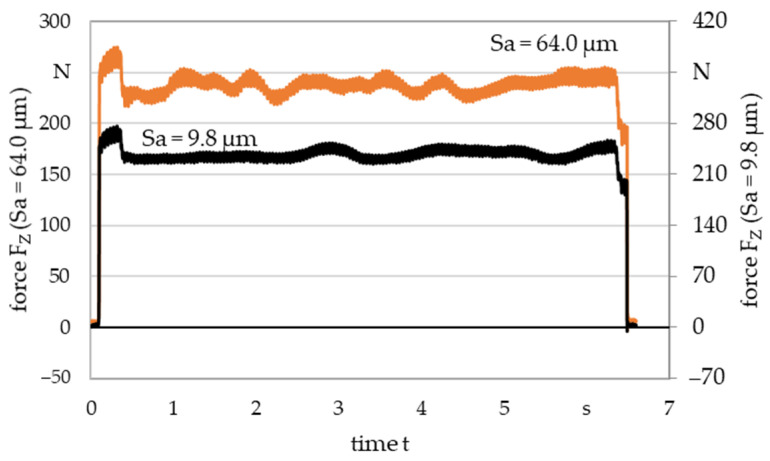
Deep-rolling force determined exemplarily while smoothing different surface roughness.

Figure 7 shows an example of the normal forces determined during deep-rolling. Shown in orange is the curve associated with specimen 19. The average values are around 242 N (cf. Section 2.4) and the curve shows an up and down movement of the force depending on the topography elevations traversed. The deep-rolled track on which the force measurement is based is indicated on the left side of Figure 6 as a red area. For comparison, the force measurement of a significantly smoother specimen (Sa = 9.8 µm, sample 1) is shown in comparison on the secondary axis in Figure 7. On average, the deep-rolling force is comparable in both cases, but the black curve shows a more constant forces level because fewer topographic elevations had to be flattened.

### 3.2. Adhesion before and after Deep-Rolling

**Figure 8:**
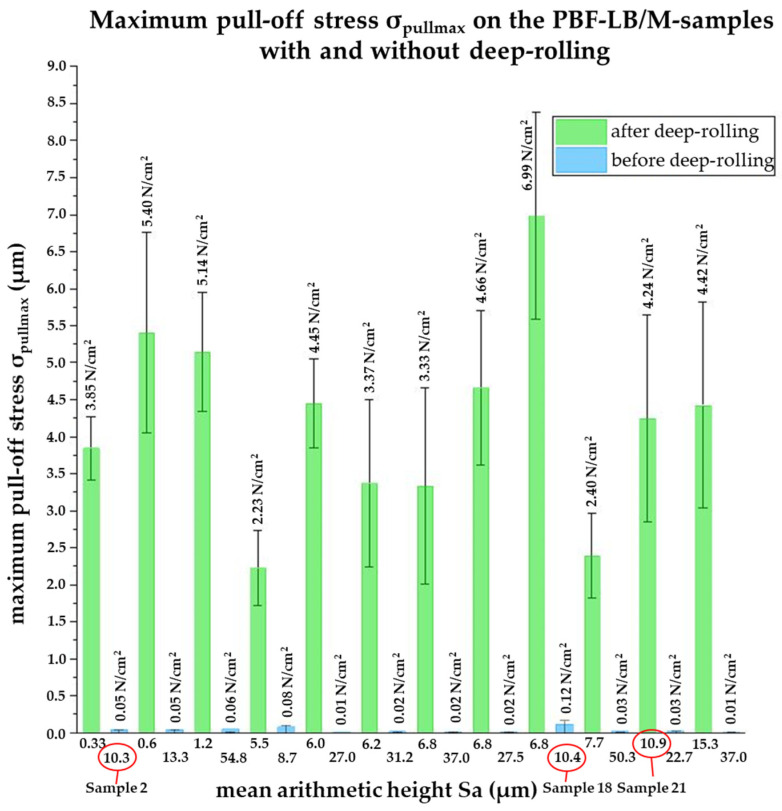
Maximum pull-off stress σ_pullmax_ on the PBF-LB/M samples with (green) and without deep-rolling (blue). The matching pairs from Table 3 are arranged next to each other.

After evaluating the microscope images, the experiments described in Section 2.3 are carried out and an average value of the pull-off stress is determined for each sample. The pull-off stress for the samples before and after deep-rolling is shown in Figure 8. The Sa values of the samples are plotted in µm and the corresponding pull-off stress in N/cm^2^. The maximum pull-off stress for the PBF-LB/M printed samples (blue) is 0.12 N/cm^2^ and the minimum pull-off stress is 0.01 N/cm^2^. In addition, the pairs of samples that belong together are arranged next to each other. In Figure 8, the maximum pull-off stress for the PBF-LB/M printed and deep-rolled samples (green) is 7 N/cm^2^ and the minimum pull-off stress is 2.2 N/cm^2^.

## 4. Discussion

**Figure 9:**
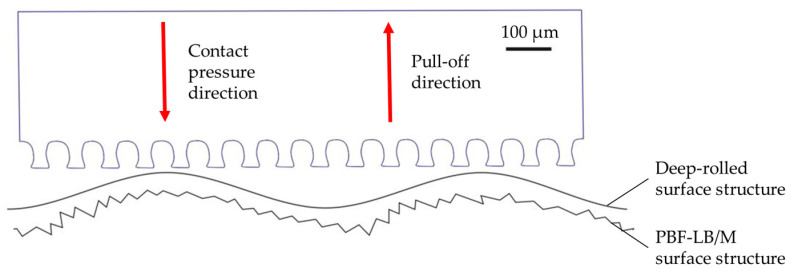
Schematic representation of the dry-adhesive microstructures versus a non-reworked surface topography and deep-rolled surface topography.

While the pull-off stress directly after the specimen generation (Sa values between 7.7 µm and 64 µm) is less than 0.2 N/cm^2^, pull-off stresses of up to 6.99 N/cm^2^ are resulting after deep-rolling. Accordingly, the pull-off stress is up to 35 times higher on the PBF-LB/M samples after deep-rolling than on the non-post-processed samples. This is because of the smoothing of the surface by deep-rolling which allows more individual microstructures of the adhesive tape to adhere to the surface and, therefore, increase the maximum pull-off stress [30]. This can be seen in Figure 9. There, a deep-rolled and a non-deep-rolled surface topography are schematically shown opposite the dry-adhesive microstructures.

**Figure 10:**
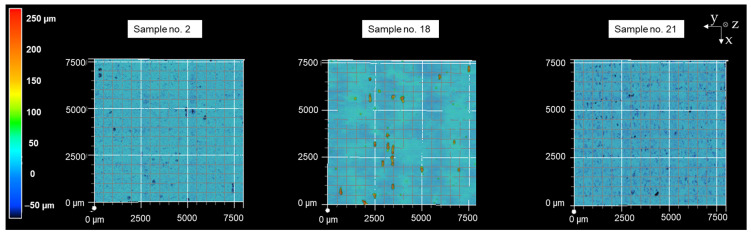
Surface topographies of samples 2, 18, and 21.

For further discussion, the samples where the Sa values before and after deep-rolling are of the same order of magnitude, but have resulted in significantly different pull-off stresses, are of particular interest. Therefore, Table 4 compares the results obtained on selected samples. While before, deep-rolling Sa values of 10.3 and 10.4 µm resulted in pull-off stresses of 0.05 and 0.12 N/cm^2^, respectively, while a slightly higher Sa value after deep-rolling of about 10.9 µm resulted in a pull-off stress of 4.24 N/cm^2^. The surface topographies from samples 2, 18, and 21 are shown in Figure 10. Samples 2 and 18 are not post-processed and sample 21 is post-processed by deep-rolling. Based on the surface structures, the smoothing of the surface by deep-rolling becomes clear. As a result, more dry-adhesive microstructures were able to form contact with the specimen surface.

**Figure 11:**
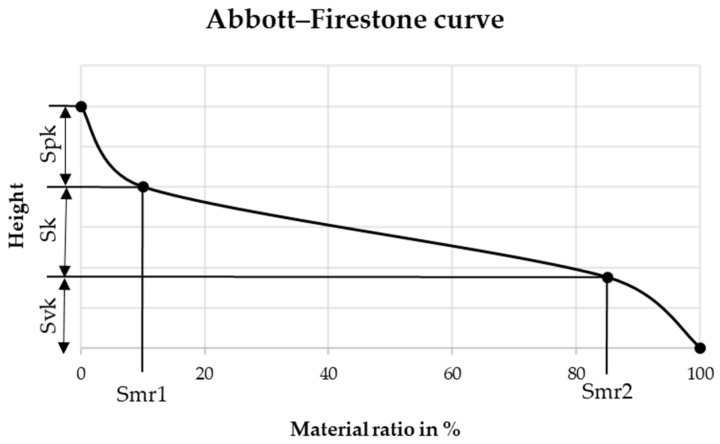
Schematic Abbott–Firestone curve.

Considering only the Sa value alone to assess the smoothing potential of deep-rolling allows an initial assessment, however, other quantities should be considered for future prediction of the adhesion of dry-adhesive microstructures. As shown in Table 4, the samples differ in their height of the core area Sk, their material fraction Smr1, as well as their material fraction Smr2 values. With the help of these parameters, the Abbott–Firestone curve, which is shown schematically in Figure 11, can be determined for the individual samples. The Abbott–Firestone curve is a graphical plot of the material ratio against the height of the profile. The material ratio describes the density of the material at the respective height. The flatter the Abbott–Firestone curve, the higher the material density in the sample surface, which means that more dry-adhesive microstructures can form contact with the surface.

The schematic diagram shows the reduced peak height Spk, the core height Sk, the reduced valley depth Svk, the peak material portion Smr1, and the valley material portion Smr2. With the help of these surface parameters, the Abbott–Firestone curve can be generated for each sample.

**Figure 12:**
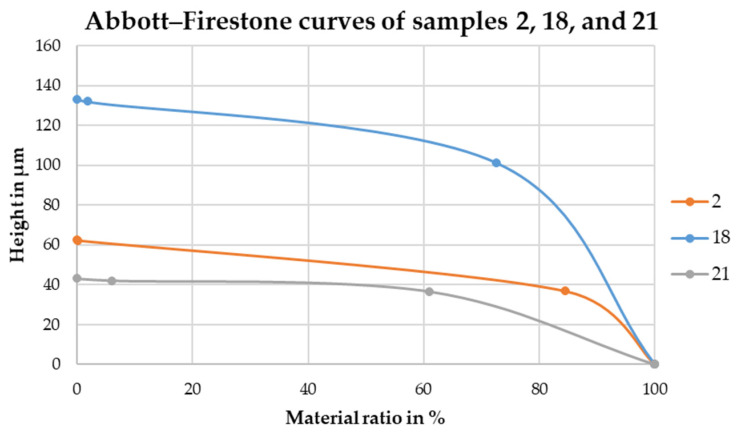
Abbott–Firestone curves of samples 2, 18, and 21.

**Figure 13:**
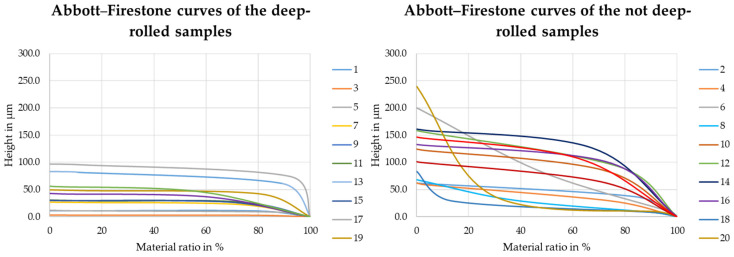
Abbott–Firestone curves of all samples.

The Abbott–Firestone curves for samples 2, 18 and 21 are shown in Figure 12 and the Abbott–Firestone curves for all samples are shown in Figure 13.

These quantities, taken from bearing area curves (also known as Abbott–Firestone curves), indicate a much flatter curve shape after deep-rolling compared to the PBF-LB/M samples, due to the lower core height and measured material fractions.

Thus, we can see the course of the bearing area curve, i.e., the shape of the surface topography influences on the adhesion of the dry-adhesive microstructures. The curve of sample 21 is significantly flatter than the curves of the samples that were not post-processed. This suggests that the material density in the sample surface is higher, which allows more individual microstructures to adhere to the surface, increasing the maximum pull-off stress [30].

This observation is confirmed by the Abbott–Firestone curves of the further samples. The curves of the deep-rolled samples are flatter and have a lower starting height compared to the curves of the non-deep-rolled samples. On the samples with the flatter Abbott–Firestone curves, more dry-adhesive microstructures can come into contact with the object surface and the maximum pull-off stress increases.

## 5. Summary and Outlook

In this work, additively manufactured samples of varied surface roughness constructed of AlSi10Mg are investigated with regard to the adhesion conditions for dry-adhesive microstructures by means of an adapted tensile test. For this purpose, dry-adhesive microstructures of dimensions 10 mm × 10 mm are pressed onto the specimen surface for 10 s with a contact force of 10 N and the pull-off stress was measured during release. It can be stated that the smoothing of the surface, as a result of an intermediate deep-rolling process, leads to an increase in the determined pull-off stress

The mean arithmetic height Sa, which is used to assess the surface quality, allows for an initial assessment of the smoothing result after deep-rolling. The parameters determined in this work allow the questions posed at the beginning to be answered and initial conclusions to be drawn with regard to the perspective processing and handling of sintered samples of larger dimensions comprised of regolith:

- Adhesion of the used dry-adhesive microstructures on the PBF-LB/M samples could be determined. The maximum pull-off stress was 0.12 N/cm^2^;
- The determined pull-off stresses show an increase by a factor of 26.41 to 392.94 after deep-rolling. Handling based on the adhesion of the dry-adhesive microstructure on a deep-rolled surface is, therefore, possible;
- The Abbott–Firestone curves of the deep-rolled specimens are much flatter and have a lower starting height compared to the curves of the non-deep-rolled specimens, allowing more dry-adhesive microstructures to form contact with the specimen surface;
- On Mars, dry-adhesive microstructures will be used to handle sintered sheets of regolith with a diameter of up to 68 mm and a maximum height of 5 mm. The diameter and height of the sheets are determined by the dimensions of the planned demonstrator. Assuming that the surface roughness and quality are comparable to that of the AlSi10Mg used in this paper, at least 1/8 of the surface must be deep-rolled, because the mass of the regolith sheet is about 64 g with a diameter of 68 mm and a height of 5 mm. For handling with a safety factor a pull-off stress of 1 N is required, on the total gripping area of 3.64 cm^2^ at least 8 N could be generated in the deep-rolled condition if the lowest pull-off stress measured in the experiments is assumed. It is also crucial where the deep-rolled surface is located on the sheet so that no shear forces act on the dry-adhesive microstructures during gripping and handling. The determination of the needed pull-off stress and, therefore, the necessary location and size of the area on the specimen also allows for an optimized design for the demonstrator regarding energy and space efficiency

In order to develop a demonstrator within the framework of subsequent work that allows the process chain shown schematically in Figure 1 to be implemented, the following aspects are the focus of further investigations:

(a) Generation and processing of sintered specimens

The next stages of the work will investigate samples from artificially contaminated materials to the level of materials obtained electrochemically from regolith, as well as the effect of different compositions and sintering regimes on surface properties. It can be assumed that the material properties of sintered samples comprised of the material investigated in this work (AlSi10Mg) slightly differ in their surface and subsurface properties from those produced by means of PBF-LM/B. Therefore, investigations on the possible influence of (deep-) rolling as well as on the handling by means of the dry-adhesive microstructure have to be carried out systematically in dependence on the sample composition.

(b) Systematic study of the adhesion conditions

In order to further understand the adhesion behavior of the dry-adhesive microstructures, experiments with different materials and manufacturing processes of the samples are carried out in the following. In addition, the wear and contamination behavior will be analyzed.

## Figures and Tables

**Table 2:** Deep-rolling parameter and force measurement settings.

Parameter	Value
tool diameter d_b_	6.35 mm
deep-rolling pressure p_dr_	10 MPa
deep-rolling force F_r_	242.33 N ± 5.97 N
step over so	0.1 mm
rolling speed v_r_	100 mm/min
lubricant	8%-emulsion
size of deep-rolled area f_a_	10 × 10 mm^2^
low pass filter L_p_	300 Hz
sampling rate s_r_	1 kHz

**Table 3:** Mean arithmetic height of the 24 samples.

Sample No.	Laser Power P, Hatch Distance D_h_, Scan Speed V_s_	Mean Arithmetic Height Sa before Deep-Rolling	Mean Arithmetic Height Sa after Deep-Rolling	Roughness Reduction ΔSa
1	100 W, 250 µm, 1500 mm/s	9.8 µm	0.3 µm	97%
2	100 W, 350 µm, 1500 mm/s	10.3 µm		
3	100 W, 350 µm, 2000 mm/s	7.7 µm	0.6 µm	92%
4	370 W, 350 µm, 1000 mm/s	13.3 µm		
5	370 W, 350 µm, 1500 mm/s	50.8 µm	1.2 µm	98%
6	370 W, 350 µm, 2000 mm/s	54.8 µm		
7	200 W, 250 µm, 1500 mm/s	57.3 µm	5.5 µm	90%
8	100 W, 250 µm, 2000 mm/s	8.7 µm		
9	200 W, 350 µm, 1500 mm/s	17.8 µm	6.0 µm	66%
10	100 W, 150 µm, 1500 mm/s	27.0 µm		
11	200 W, 250 µm, 2000 mm/s	31.6 µm	6.2 µm	80%
12	200 W, 250 µm, 1000 mm/s	31.2 µm		
13	300 W, 250 µm, 1000 mm/s	20.5 µm	6.8 µm	67%
14	200 W, 350 µm, 2000 mm/s	37.0 µm		
15	200 W, 350 µm, 1000 mm/s	23.5 µm	6.8 µm	71%
16	100 W, 350 µm, 1000 mm/s	27.5 µm		
17	300 W, 350 µm, 1000 mm/s	60.6 µm	6.8 µm	89%
18	100 W, 350 µm, 1500 mm/s	10.4 µm		
19	100 W, 250 µm, 1000 mm/s	64.0 µm	7.7 µm	88%
20	100 W, 350 µm, 1000 mm/s	50.3 µm		
21	300 W, 250 µm, 1500 mm/s	23.8 µm	10.9 µm	54%
22	300 W, 350 µm, 1500 mm/s	22.7 µm		
23	200 W, 350 µm, 1000 mm/s	32.0 µm	15.3 µm	52%
24	200 W, 250 µm, 2000 mm/s	37.0 µm		

**Table 4:** Samples with comparable Sa values (gray background: deep-rolled samples).

Sample No.	2	18	21
**Mean arithmetic height Sa**	10.3 µm	10.4 µm	10.9 µm
**Pull-off stress σ_pullmax_**	0.05 N/cm^2^	0.12 N/cm^2^	4.24 N/cm^2^
**Height of the core area Sk**	25.32 µm	30.64 µm	5.50 µm
**Material fraction Smr1**	0.18%	1.91%	6.11%
**Material fraction Smr2**	84.53%	72.61%	61.08%
**Arithmetic average roughness Ra**	10.3 µm	10.4 µm	10.9 µm
**Mean roughness depth Rz**	367.0 µm	262.5 µm	502.5 µm
**Maximum height of the ripple Wz**	214.7 µm	203.9 µm	366.3 µm

## Data Availability

The data presented in this study are available on request from the corresponding author.
